# Hypocoagulability and Platelet Dysfunction Are Exacerbated by Synthetic Colloids in a Canine Hemorrhagic Shock Model

**DOI:** 10.3389/fvets.2018.00279

**Published:** 2018-11-13

**Authors:** Corrin J. Boyd, Melissa A. Claus, Anthea L. Raisis, Giselle Hosgood, Claire R. Sharp, Lisa Smart

**Affiliations:** School of Veterinary and Life Sciences, College of Veterinary Medicine, Murdoch University Perth, WA, Australia

**Keywords:** hydroxyethyl starch, succinylated gelatin, crystalloid, fresh whole blood, platelet closure time, PFA-100, rotational thromboelastometry (ROTEM), viscoelastic coagulation tests

## Abstract

**Background:** Hemorrhagic shock and volume replacement can alter coagulation. Synthetic colloids, hydroxyethyl starch (HES), and gelatin, may enhance hypocoagulability. Our primary objective was to describe the effect of four fluid products on coagulation in canine hemorrhagic shock. Our secondary objective was to compare measurements of coagulation during shock to baseline in all dogs.

**Methods:** Anesthetized greyhounds subjected to atraumatic hemorrhage for 60 min were administered 20 mL kg^−1^ of either fresh whole blood (FWB), 6% HES 130/0.4, 4% succinylated gelatin (GELO), or 80 mL kg^−1^ of isotonic crystalloid over 20 min (*n* = 6 per group). Platelet closure time (PCT), rotational thromboelastometry (ROTEM) and plasma coagulation assays were measured at baseline, end of hemorrhage (shock), and 40 (T60), and 160 (T180) min after study fluid. ROTEM parameters included clotting time (CT), clot formation time (CFT), alpha angle, maximum clot firmness (MCF), lysis index at 60 min (LI60), and thrombodynamic potential index (TPI) for INTEM, EXTEM, FIBTEM (MCF only), and APTEM (LI60 only) profiles. Plasma coagulation assays included prothrombin time (PT), activated partial thromboplastin time (APTT), fibrinogen concentration and activities of factor VII (FVII), factor VIII (FVIII), and von Willebrand Factor antigen (vWF). Between-group differences were tested using linear mixed models with *post-hoc* between-group comparisons (Bonferroni-Holm corrected). Differences between baseline and shock were tested using paired *t-*tests. Significance was set at *P* < 0.05.

**Results:** GELO showed longer PCT at T60, compared with FWB and CRYST, and at T180, compared with all other groups. HES showed longer EXTEM CT at T60, compared with all other groups. HES showed lower INTEM and EXTEM MCF at T60 and lower INTEM MCF at T180, compared with FWB. Some plasma coagulation assays showed greater hypocoagulability with HES. Comparing shock to baseline, EXTEM CT, INTEM CFT, EXTEM CFT, PT, and FVIII significantly increased and PCT, INTEM CT, INTEM MCF, EXTEM MCF, EXTEM LI60, EXTEM TPI, FIBTEM MCF, APTT, fibrinogen, FVII, and vWF significantly decreased.

**Conclusions:** In dogs with hemorrhagic shock, volume replacement with GELO caused mild platelet dysfunction and HES was associated with coagulation changes consistent with hypocoagulability, beyond effects of hemodilution. Shock alone produced some evidence of hypocoagulability.

## Introduction

Hemorrhagic shock in dogs, due to traumatic, surgical, or spontaneous hemorrhage, can result in organ dysfunction and death (1–3). Hypocoagulability is a potential complication of shock that can perpetuate hemorrhage. Clinical studies have shown evidence of both hypocoagulability and hypercoagulability in dogs experiencing hemorrhage (4–8), however results of clinical studies can be difficult to interpret due to patient heterogeneity and temporal variability of blood sampling in relation to injury and treatment. There are limited experimental studies examining coagulation during atraumatic hemorrhagic shock in dogs, however, one study demonstrated decreased platelet aggregation and mild hypocoagulability (9), while another showed no significant change in platelet function (10).

Treatment of hemorrhagic shock includes restoration of circulating blood volume by administration of intravenous crystalloid fluids, synthetic colloid fluids, or blood products. Synthetic colloids such as hydroxyethyl starch (HES) may be chosen by veterinarians (11) due to their potentially superior volume-expanding ability compared with crystalloids (12), as well as their lower cost and greater availability compared with blood products. However, some synthetic colloids have been associated with hypocoagulability, which may increase risk of bleeding in a patient already experiencing perturbed hemostasis. In dogs, studies including *in vitro* (13–17), *in vivo* healthy dog (18, 19), and experimental sepsis (20) designs have shown an association between HES and both decreased platelet function and hypocoagulability. Only one study has compared the effects of low molecular weight (MW) HES with isotonic crystalloid under the conditions of atraumatic hemorrhagic shock (10). This study showed no difference in platelet function beyond the effects of hemodilution, as measured by platelet closure time (PCT), however other tests of coagulation were not performed. Given the risk of potentiating hypocoagulability in clinical patients, further information about the coagulation effects of HES administration in dogs with hemorrhagic shock is needed.

Succinylated gelatins are a group of synthetic colloids that are widely available in Europe and Australasia (11), and may provide an alternative to HES products. However, there is limited evidence for their safety profile, in both human and veterinary medicine. Gelatin colloids have been associated with hypocoagulability in humans both *in vitro* (21, 22) and *in vivo* (23). *In vitro* dilution studies comparing gelatin to HES colloids have shown greater hypocoagulability with HES (21, 22, 24), suggesting gelatin may be the preferred colloid. The effects of gelatin colloids on coagulation in dogs have not been thoroughly investigated. One study showed decreased von Willebrand Factor antigen (vWF), platelet count and fibrinogen concentration following oxypolygelatin administration to healthy dogs (25). However, these changes may have been due to hemodilution, as there was no crystalloid control.

Our study aimed to assess the effects of four different intravenous fluid products on PCT, rotational thromboelastometry (ROTEM), and plasma coagulation assays in a canine experimental model of atraumatic hemorrhage under general anesthesia. We compared the effects of 6% HES 130/0.4, 4% succinylated gelatin (GELO), fresh whole blood (FWB) and balanced isotonic crystalloid (CRYST). In our study, FWB served as a natural colloid comparison and CRYST served as a hemodilution comparison. We hypothesized that volume replacement with HES or GELO would result in the longest PCT and most marked evidence of hypocoagulability, followed by CRYST, then FWB. As a secondary exploratory objective, we compared the effects of hemorrhagic shock alone on the above measures of coagulation in all subjects. We hypothesized that hemorrhagic shock, prior to fluid resuscitation, would be associated with decreased PCT and hypocoagulability.

## Materials and methods

### Animals

This study was carried out in accordance with the recommendations of the Australian Code for the Care and Use of Animals for Scientific Purposes. The protocol was approved by the Murdoch University Animal Ethics Committee (R2666/14). This work represents part of a larger study that also included assessment of glycocalyx shedding and inflammation (26), and acute kidney injury after fluid resuscitation. Ex-racing greyhounds that were unable to be rehomed were used. Dogs were deemed healthy based on assessment of physical examination, basic clinicopathologic parameters (packed cell volume (PCV), total plasma protein, platelet count, urinalysis), and ultrasound of the kidneys and urinary bladder. In accordance with animal ethics requirements, blood removed during the experiment that was not re-infused was made available to a veterinary hospital blood bank, therefore blood bank stock levels influenced study fluid allocation and precluded randomization. Dogs were housed overnight in a kennel environment with free access to water before the day of the experiment.

### Anesthesia and instrumentation

Dogs were premedicated with 0.3 mg kg^−1^ methadone IM. Thirty minutes later, anesthesia was induced with alfaxalone, titrated up to 3 mg kg^−1^ via a cephalic venous cannula. Dogs were orotracheally intubated and anesthesia was maintained with isoflurane delivered by a rebreathing system, titrated to achieve an end-tidal concentration of 1.4%. Dogs were mechanically ventilated with a 0.3 fraction of inspired oxygen concentration, with ventilation rate adjusted before baseline measurements to achieve an end-tidal carbon dioxide partial pressure of 35–40 mmHg. Body temperature was monitored via an esophageal thermistor probe and was maintained between 36 and 38°C by a heating mat and warmed air delivery system. Infusions of compound sodium lactate (CSL) at 10 mL kg^−1^ hr^−1^ and fentanyl at 2 μg kg^−1^ hr^−1^ were administered IV throughout the experiment, with the exception of suspending CSL during study fluid infusion. Heart rate, arterial blood pressure, pulse oximetry, end-tidal carbon dioxide and isoflurane, body temperature, and anesthetic depth (eye position, facial reflexes, and jaw tone) were monitored continuously and recorded at 5-min intervals throughout anesthesia.

Instrumentation was performed with the dogs positioned in left lateral recumbency. Percutaneous 0.5% bupivacaine was instilled adjacent to the femoral nerve via ultrasound guidance and a 14 G, 88 mm cannula was inserted into the femoral artery after surgical exposure. This cannula was used for continuous arterial pressure monitoring, arterial blood sampling, and blood removal during the shock phase. A 14 G, 133 mm cannula was inserted percutaneously into the distal right jugular vein and advanced to terminate between the thoracic inlet and right atrium. This cannula was used for continuous central venous pressure monitoring, venous blood sampling, and study fluid administration. A urethral Foley catheter was placed for urine output monitoring and to collect samples for the acute kidney injury arm of the project. At the end of instrumentation, dogs were repositioned in dorsal recumbency. Blood pressure transducers were positioned at the approximate level of the heart base (scapulohumeral joint) and calibrated to atmospheric pressure. After repositioning and prior to baseline data collection, if mean arterial blood pressure (MAP) decreased to <60 mmHg for longer than 10 min, up to 10 mL kg^−1^ of CSL was infused until MAP increased to >60 mmHg. A minimum of 10 min elapsed after fluid infusion before baseline data and blood samples were collected.

### Experimental procedure

**Figure 1:**
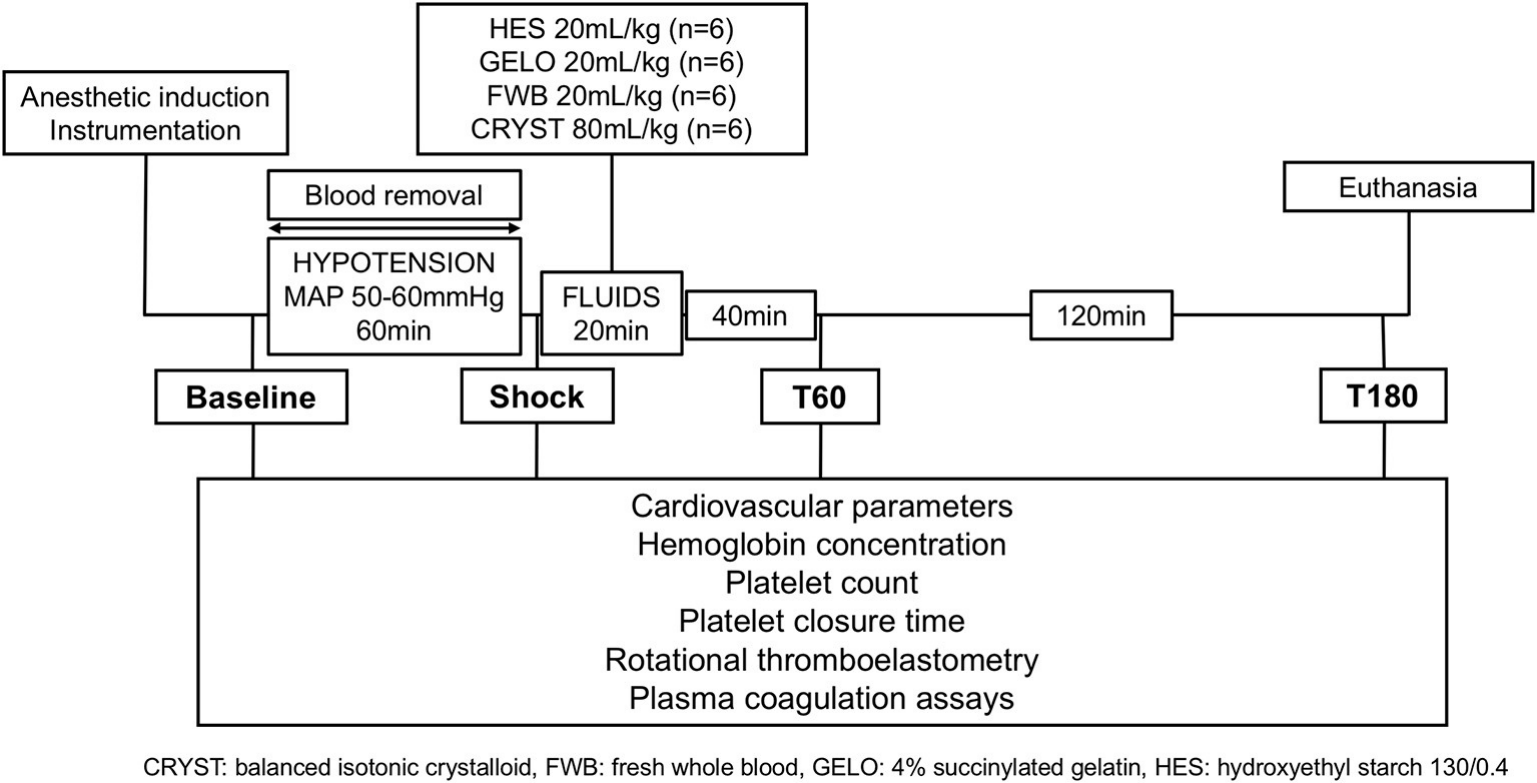
Summary of experimental design.

The experimental design is summarized in Figure 1. Baseline data and samples were collected immediately before blood removal, which was achieved by passive flow from the femoral arterial catheter into blood collection bags, containing citrate-phosphate-dextrose-adenine anticoagulant. Once a MAP of 50 mmHg was reached, blood flow into collection bags was stopped. Blood was then intermittently removed via a 60 mL syringe to maintain MAP of 50–60 mmHg for 60 min. The total volume of blood removed was calculated from change in weight of collection bags and volume removed by syringe. Blood collection bags were stored at room temperature until they were either re-infused into the dog (FWB group) or transferred to the blood bank.

At the end of the 60-min hypotension phase (shock), dogs were administered one of four study fluids IV over 20 min including; 20 mL kg^−1^ of autologous FWB, balanced HES 130/0.4 (Volulyte 6%, Fresenius Kabi) or GELO (Gelofusine, B. Braun Medical), or 80 mL kg^−1^ of CRYST (Plasmalyte-148, Baxter Healthcare) (*n* = 6, per group). Previous work has shown that these volumes of crystalloid and HES given to greyhounds in hemorrhagic shock have produced similar effects on oxygen extraction ratio and levels of hemodilution within 60 min of bolus (27). Dogs were then monitored for a further 3 h before euthanasia with IV pentobarbitone 150 mg kg^−1^. Cadavers were retained for other teaching and research purposes within the university.

### Data measurement and sample collection

Cardiovascular variables (heart rate, arterial and central venous pressure, pulse pressure variation, and cardiac output) were recorded at baseline, after 60 min of hypotension (shock), and then 40 (T60) and 160 (T180) min after the end of fluid administration (Figure 1). Cardiac output was measured by the lithium dilution technique, as previously described (27). Arterial and venous blood samples were collected for measurement of coagulation parameters, blood gas, electrolytes, lactate, and hemoglobin at the same time points. Blood samples for blood gas, electrolytes, and lactate were collected anaerobically into heparinized syringes and stored on ice until analysis within 10 min. Calculated variables included body surface area and oxygen extraction ratio, using equations previously described (27).

### Coagulation parameters

Blood for PCT, ROTEM, and plasma coagulation assays was collected by syringe from the femoral arterial cannula and transferred into blood tubes containing 3.2% buffered sodium citrate with an anticoagulant:blood ratio of 1:9 (final concentration 10.8 mM citrate). Tubes were gently agitated to ensure even mixing of anticoagulant. Blood for PCT and ROTEM was stored at room temperature until analysis. Blood for plasma coagulation assays was stored on ice for <1 h, centrifuged at 1,350 × g for 10 min, the supernatant separated into aliquots and then stored at −80°C for later batched analysis.

Platelet closure time was measured in duplicate within 10 min of sample collection using the Platelet Function Analyzer-100 (Dade Boehring Inc.) and collagen and adenosine diphosphate cartridges, as previously described (10). Analysis was immediately repeated if the coefficient of variation was >15%. Platelet count was estimated on Diff-Quik stained smears by manually counting platelets in 10 high-power fields and multiplying the average number of platelets per field by 15 to convert to × 10^9^ L^−1^.

Rotational thromboelastometry (ROTEM^®;^ *delta*, Tem International GmbH) was performed according to the manufacturer's instructions and PROVETS guidelines (28) using the INTEM (star-TEM and in-TEM reagents), EXTEM (star-TEM and r ex-TEM reagents), FIBTEM (r ex-TEM and fib-TEM reagents), and APTEM (r ex-TEM and ap-TEM reagents) profiles. Measurement was started 30 min after sample collection. Each profile was run for at least 1 h following initiation. Data recorded for the INTEM and EXTEM profiles included clotting time (CT), clot formation time (CFT), alpha angle (α), maximum clot firmness (MCF), and lysis index at 60 min (LI60). Thrombodynamic potential index (TPI) was recorded for the EXTEM profile as a measure of global coagulation (29), calculated using the equations:

$$\begin{array}{l} \text{ TPI}=\text{EMX}*30/\text{CFT},\\ \text{EMX}=\left(100*\text{MCF}\right)/\left(100-\text{MCF}\right). \end{array}$$

Data recorded for the FIBTEM profile included MCF. Hypocoagulability was defined as at least one of: prolonged CT or CFT, reduced α, MCF, or TPI. Hypercoagulability was defined as at least one of: shortened CT or CFT, elevated α, MCF or TPI. Hyperfibrinolysis was assessed by comparing the EXTEM and APTEM profiles and scored with a novel (unvalidated) system, assigning samples to one of four categories based on the authors' interpretation: no hyperfibrinolysis, defined as LI60 >90% on both EXTEM and APTEM; reversible hyperfibrinolysis, defined as LI60 <90% on EXTEM and >90% on APTEM; irreversible hyperfibrinolysis, defined as LI60 <90% on both EXTEM and APTEM; and paradoxical increase in hyperfibrinolysis, defined as LI60 >90% on EXTEM and <90% on APTEM.

Plasma coagulation assays, including prothrombin time (PT), activated partial thromboplastin time (APTT), fibrinogen concentration, activity of factor VII (FVII) and factor VIII (FVIII), and vWF, were performed using a commercial coagulation analyser (ACL Top 300, Beckman Coulter). All assays were performed according to the manufacturer's instructions. Commercially-available human factor-deficient plasma was used for the measurement of FVII and FVIII. Results of FVII, FVIII, and vWF were compared with a pooled sample of all baseline plasma from dogs in this study.

### Statistical methods

Visualization of histograms was used to confirm normal distributions of continuous data, including baseline characteristics, cardiovascular parameters and coagulation parameters, and are summarized as mean (95% confidence interval). Analysis of differences in baseline characteristics and cardiovascular data between groups has been previously described (26). Differences between groups in variables measured over time were tested using mixed effect linear models. Significant time-treatment interactions prompted *post-hoc* between-group comparison of least-square means. Differences in the frequency of hyperfibrinolysis scores between groups were tested using Fisher's exact test. Differences between baseline and shock in all dogs, as per the secondary objective of this study, were tested using paired Student's *t-*tests. Significance was set at *P* ≤ 0.05. *Post-hoc* pairwise comparisons were considered significant against a Bonferroni–Holm adjusted significance level to control for Type I error (30). Data were analyzed using SAS Version 9.4 (SAS Institute).

## Results

### Baseline and cardiovascular data

Twenty-seven ex-racing greyhounds (23 male, 4 female) were used. Three dogs (2 male, 1 female) were excluded due to equipment malfunction at the start of, or during, the experiment, and data is presented for the remaining 24. Baseline and cardiovascular parameters have been previously reported (26) and only major findings are summarized below. There were no significant between-group differences in age (all dogs, range 1.6–4.0 years), sex, body weight (all dogs, range 27.7–35.0 kg), body surface area (all dogs, range 0.93–1.13 m^2^), blood volume removed (all dogs, range 35–66 mL kg^−1^), or baseline cardiovascular parameters. Two dogs received additional CSL for hypotension prior to baseline: 6.8 mL kg^−1^, GELO group and 2.2 mL kg^−1^, CRYST group. There were some differences in measures of shock between groups directly after fluid administration, but these differences resolved by T60. The only differences in hemoglobin were between the FWB group and all other groups, as expected (26).

### Comparisons between fluid groups

There were no differences between groups in any coagulation parameters at baseline or shock (all *P* > 0.05). Several between-group differences were found after fluid administration.

There was evidence of platelet dysfunction in the GELO group (Table 1). Platelet closure time was longer at T60 in the GELO group, compared with FWB (*P* < 0.001) and CRYST (*p* = 0.003). Platelet closure time was also longer at T180 in the GELO group, compared with all other groups (HES *P* = 0.001, FWB *P* < 0.001, CRYST *P* < 0.001). There were no significant differences between any of the groups in estimated platelet count.

**Table 1:** Platelet closure time and count (mean, 95% confidence interval) in dogs (*n* = 6 per group) with hemorrhagic shock given 20 mL kg^−1^ of either fresh whole blood (FWB), hydroxyethyl starch 130/0.4 (HES), 4% succinylated gelatin (GELO), or 80 mL kg^−1^ of balanced isotonic crystalloid (CRYST).

**Parameter**	**Baseline**	**Shock**	**T60**	**T180**
**PLATELET CLOSURE TIME (SEC)**				
FWB	79.2 (65.5–92.8)	65.7 (54.7–76.6)	72.7 (64.1–81.3)^#^	73.3 (63.7–83.0)^#^
CRYST	92.9 (75.5–110.4)	72.6 (61.7–83.5)	85.3 (78.8–91.8)^#^	77.9 (64.3–91.5)^#^
HES	83.2 (76.9–89.4)	65.8 (56.2–75.3)	93.1 (81.1–105.1)	84.7 (64.4–104.9)^#^
GELO	78.3 (63.2–93.3)	72.0 (66.9–77.1)	111.1 (84.0–138.2)^&^, *	113.0 (82.0–144.0)^&^, *, ^+^
**ESTIMATED PLATELET COUNT (X10**^9^**/L**^−1^**)**				
FWB	144.0 (122.6–165.4)	131.3 (74.6–187.9)	127.5 (94.9–160.1)	144.0 (91.7–196.3)
CRYST	126.8 (95.5–158.0)	112.5 (91.8–133.2)	67.9 (21.5–114.3)	78.3 (60.0–96.5)
HES	133.8 (110.7–156.8)	115.1 (65.7–164.6)	104.3 (67.9–140.6)	88.3 (50.7–125.8)
GELO	134.3 (113.1–155.4)	117.0 (105.5–128.5)	135.5 (94.4–176.6)	105.8 (76.3–135.2)

*Fluid was delivered directly after shock time point*.

& Significantly different to FWB;

* Significantly different to CRYST;

+ Significantly different to HES;

# *Significantly different to GELO (Bonferroni–Holm corrected P-value < 0.05)*.

Rotational thromboelastometry showed some evidence of hypocoagulability in the HES group (Table 2). INTEM MCF was lower with HES at T60 (*P* = 0.002) and T180 (*P* = 0.008), compared with FWB. EXTEM CT was longer at T60 in the HES group, compared with all other groups (FWB *P* < 0.001, CRYST *P* = 0.002, GELO *P* = 0.004). EXTEM MCF was lower at T60 in the HES group, compared with FWB (*P* < 0.001). Otherwise, there were no significant differences in ROTEM parameters between any of the groups. No between-group differences were detected in hyperfibrinolysis scores (Table 3).

**Table 2:** Rotational thromboelastometry parameters (mean, 95% confidence interval) in dogs (*n* = 6 per group) with hemorrhagic shock given 20 mL kg^−1^ of either fresh whole blood (FWB), hydroxyethyl starch 130/0.4 (HES), 4% succinylated gelatin (GELO), or 80 mL kg^−1^ of balanced isotonic crystalloid (CRYST).

**Parameter**	**Baseline**	**Shock**	**T60**	**T180**
**INTEM CT (sec)**				
FWB	279.3 (137.5–421.2)	204.8 (123.1–286.5)	330.5 (17.2–643.8)	269.0 (150.7–387.3)
CRYST	171.3 (149.1–193.5)	160.7 (129.2–192.1)	162.2 (134.0–190.3)	159.8 (142.8–176.9)
HES	201.2 (129.1–273.2)	145.8 (72.2–219.5)	231.8 (130.4–333.3)	199.0 (113.0–285.0)
GELO	221.5 (146.0–297.0)	161.0 (117.0–205.0)	224.3 (108.6–340.1)	193.5 (146.5–240.5)
**INTEM CFT (sec)**				
FWB	224.2 (143.1–305.2)	260.3 (180.6–340.0)	303.8 (102.0–505.7)	235.3 (178.9–291.8)
CRYST	143.5 (119.1–167.9)	227.8 (190.9–264.8)	298.8 (269.5–328.1)	234.5 (207.6–261.4)
HES	202.8 (106.9–298.8)	264.0 (168.0–360.0)	431.5 (287.3–575.7)	309.2 (221.0–397.3)
GELO	178.2 (128.4–227.9)	186.2 (114.2–258.1)	305.5 (211.1–399.9)	278.7 (178.1–379.2)
**INTEM** **α** **(°)**				
FWB	54.2 (44.9–63.4)	54.0 (46.3–61.7)	52.7 (38.5–66.8)	56.2 (49.8–62.6)
CRYST	68.0 (65.4–70.6)	62.7 (57.2–68.1)	56.8 (53.7–60.0)	60.7 (59.8–61.5)
HES	60.5 (49.4–71.6)	56.0 (46.5–65.5)	50.7 (40.2–61.1)	53.5 (46.4–60.6)
GELO	61.2 (56.5–65.9)	61.7 (53.8–69.6)	53.0 (46.7–59.3)	56.2 (49.8–62.6)
**INTEM MCF (mm)**				
FWB	51.2 (46.9–55.4)	47.7 (43.0–52.3)	45.3 (38.3–52.3)^+^	48.2 (44.5–51.8)^+^
CRYST	54.2 (50.8–57.5)	46.3 (43.0–49.7)	42.2 (39.9–44.4)	44.0 (41.4–46.6)
HES	50.3 (45.3–55.4)	44.5 (40.3–48.7)	37.0 (31.6–42.4)^&^	41.0 (36.2–45.8)^&^
GELO	54.0 (48.3–59.7)	51.2 (45.8–56.5)	41.5 (36.0–47.0)	42.8 (37.2–48.5)
**INTEM LI60 (%)**				
FWB	99.0 (97.4–100.6)	99.7 (98.8–100.5)	99.7 (98.8–100.5)	99.2 (97.9–100.4)
CRYST	99.7 (98.8–100.5)	98.7 (97.6–99.8)	96.8 (94.7–99.0)	97.2 (94.4–99.9)
HES	99.2 (97.9–100.4)	98.5 (96.4–100.6)	87.8 (67.3–108.4)	90.0 (74.1–105.9)
GELO	99.5 (98.6–100.4)	99.7 (99.1–100.2)	98.3 (94.0–102.6)	96.5 (87.5–105.5)
**EXTEM CT (sec)**				
FWB	52.5 (40.8–64.2)	75.2 (57.2–93.1)	64.7 (45.3–84.1)^+^	62.0 (53.1–70.9)
CRYST	54.8 (37.8–71.9)	66.2 (59.4–72.9)	72.8 (62.9–82.8)^+^	71.5 (58.7–84.3)
HES	61.8 (40.6–83.0)	69.0 (56.1–81.9)	119.3 (57.4–181.3)^&^^,^^*^^,^^#^	82.7 (50.6–114.8)
GELO	47.8 (28.6–67.0)	55.8 (36.1–75.6)	76.2 (27.5–124.8)^+^	75.2 (50.6–99.8)
**EXTEM CFT (sec)**				
FWB	178.2 (121.5–234.8)	217.3 (149.5–285.2)	251.0 (127.6–374.4)	202.2 (148.5–255.9)
CRYST	129.3 (115.4–143.3)	184.2 (152.6–215.7)	246.0 (198.3–293.7)	221.0 (177.7–264.3)
HES	187.2 (106.0–268.3)	208.0 (137.8–278.2)	373.4 (179.9–566.9)	437.3 (0.0–918.6)
GELO	139.3 (102.6–176.1)	165.0 (115.5–214.5)	230.4 (167.7–293.1)	263.3 (174.5–352.1)
**EXTEM** **α** **(°)**				
FWB	59.8 (53.5–66.1)	60.0 (55.9–64.1)	55.8 (48.8–62.9)	58.3 (54.4–62.3)
CRYST	66.5 (64.4–68.6)	63.3 (60.9–65.8)	59.3 (54.2–64.5)	60.8 (59.0–62.6)
HES	61.7 (55.1–68.2)	61.8 (56.4–67.2)	46.2 (28.6–63.8)	53.0 (39.8–66.2)
GELO	65.7 (62.8–68.5)	63.5 (59.9–67.1)	49.5 (32.7–66.3)	56.0 (52.5–59.5)
**EXTEM MCF (mm)**				
FWB	50.5 (46.3–54.7)	44.7 (39.6–49.7)	45.3 (38.6–52.1)^+^	47.0 (43.4–50.6)
CRYST	54.7 (52.1–57.2)	45.7 (42.1–49.3)	39.0 (33.3–44.7)	39.3 (34.7–44.0)
HES	49.3 (42.7–55.9)	42.5 (33.4–51.6)	31.2 (20.5–41.9)^&^	37.8 (28.6–47.1)
GELO	54.3 (47.8–60.8)	50.0 (43.4–56.6)ß	39.0 (26.6–51.4)	40.3 (31.9–48.8)
**EXTEM TPI**				
FWB	18.8 (12.7–25.0)	12.5 (6.7–18.3)	12.3 (5.6–19.0)	14.0 (9.6–18.4)
CRYST	28.5 (22.8–34.2)	14.3 (10.5–18.2)	8.2 (5.6–10.8)	9.3 (6.1–12.6)
HES	20.2 (7.4–32.9)	14.2 (1.1–27.3)	4.8 (2.0–7.6)	8.2 (1.4–14.9)
GELO	28.8 (15.3–42.4)	21.0 (10.3–31.7)	11.2 (5.3–17.1)	9.5 (4.2–14.8)
**EXTEM LI60 (%)**				
FWB	89.3 (66.9–111.7)	78.7 (48.1–109.3)	90.7 (70.7–110.7)	91.0 (78.2–103.8)
CRYST	99.0 (97.9–100.1)	87.7 (73.0–102.3)	84.7 (52.9–116.5)	78.8 (58.5–99.2)
HES	98.3 (95.5–101.2)	83.3 (56.1–110.6)	74.3 (36.5–112.2)	84.3 (56.2–112.5)
GELO	97.5 (93.8–101.2)	86.7 (65.3–108.1)	75.8 (49.2–102.5)	72.5 (42.5–102.5)
**FIBTEM MCF (mm)**				
FWB	4.3 (3.5–5.2)	3.5 (2.4–4.6)	3.3 (1.5–5.2)	2.8 (1.0–4.6)
CRYST	6.0 (3.1–8.9)	4.7 (1.9–7.5)	2.8 (1.0–4.6)	3.2 (1.2–5.1)
HES	5.0 (2.6–7.4)	2.0 (0.0–4.9)	0.7 (0.0–2.4)	2.2 (0.0–5.2)
GELO	4.8 (3.3–6.4)	3.8 (2.3–5.4)	2.0 (0.2–3.8)	2.7 (1.1–4.2)

*Fluid was delivered directly after Shock time point*.

& *Significantly different to FWB*;

* *Significantly different to CRYST*;

+ *Significantly different to HES*;

# *Significantly different to GELO (Bonferroni–Holm corrected P-value < 0.05). α, alpha angle; CFT, clot formation time; CT, clotting time; LI60, lysis index at 60 min; MCF, maximum clot firmness; TPI, thrombodynamic potential index*.

**Table 3:** Hyperfibrinolysis scores in dogs (*n* = 6 per group) with hemorrhagic shock given 20 mL kg^−1^ of either fresh whole blood (FWB), hydroxyethyl starch 130/0.4 (HES), 4% succinylated gelatin (GELO), or 80 mL kg^−1^ of balanced isotonic crystalloid (CRYST).

**Group**	**Baseline**	**Shock**	**T60**	**T180**
HES	6, 0, 0, 0	4, 1, 1, 0	3, 1, 2, 0	4, 1, 1, 0
GELO	6, 0, 0, 0	4, 2, 0, 0	2, 3, 1, 0	3, 1, 2, 0
FWB	5, 0, 1, 0	3, 0, 2, 1	4, 0, 1, 1	4, 1, 1, 0
CRYST	6, 0, 0, 0	4, 1, 1, 0	4, 0, 2, 0	2, 1, 3, 0
*P-*value*	1.00	0.83	0.50	0.97

*Fluid was delivered directly after shock time point. Data represent the number of dogs assigned to the categories of no hyperfibrinolysis, reversible hyperfibrinolysis, irreversible hyperfibrinolysis, or paradoxical increase in hyperfibrinolysis, respectively*.

* *Fisher's exact test*.

Plasma coagulation assays showed evidence of hypocoagulability in all other groups compared with FWB, with some evidence of greater hypocoagulability in the HES group (Table 4). PT was shorter in the FWB group, compared with all other groups, at T60 (all *P* < 0.001) and T180 (CRYST *P* = 0.002, HES *P* = 0.001, GELO *P* = 0.004). PT was also shorter in the GELO group at T60, compared with HES (*P* = 0.011). APTT was shorter in the FWB group at T60, compared with all other groups (all *P* < 0.001), and at T180, compared with HES (*P* = 0.002). APTT was also shorter in the GELO group at T60, compared with HES (*P* = 0.005). Fibrinogen concentration was higher in the FWB group at T60, compared with all other groups (CRYST *P* < 0.001, HES *P* < 0.001, GELO *P* = 0.012). FVII was higher in the FWB group at T60, compared with all other groups (CRYST *P* = 0.002, HES *P* < 0.001, GELO *P* < 0.001), and at T180, compared with HES (*P* = 0.005) and GELO (*P* = 0.009). FVIII was higher in the FWB group at T60, compared with all other groups (all *P* < 0.001). vWF was higher in the FWB group at T60, compared with all other groups (CRYST *P* < 0.001, HES *P* = 0.002, GELO *P* = 0.007).

**Table 4:** Plasma coagulation assay parameters (mean, 95% confidence interval) in dogs (*n* = 6 per group) with hemorrhagic shock given 20 mL kg^−1^ of either fresh whole blood (FWB), hydroxyethyl starch 130/0.4 (HES), 4% succinylated gelatin (GELO), or 80 mL kg^−1^ of balanced isotonic crystalloid (CRYST).

**Parameter**	**Baseline**	**Shock**	**T60**	**T180**
**PROTHROMBIN TIME (SEC)**				
FWB	7.5 (7.2–7.7)	8.4 (7.9–8.9)	8.3 (7.8–8.8)*,+,#	8.5 (8.1–8.9)*,+,#
CRYST	7.4 (7.2–7.7)	8.5 (8.1–8.8)	11.4 (10.2–12.7)^&^	10.3 (9.6–11.0)^&^
HES	7.8 (7.2–8.3)	8.9 (7.6–10.2)	12.0 (9.4–14.7)^&^, ^#^	10.3 (8.9–11.8)^&^
GELO	7.4 (6.9–7.9)	8.1 (7.7–8.5)	10.6 (9.5–11.6)^&^, ^+^	10.2 (9.2–11.1)^&^
**ACTIVATED PARTIAL THROMBOPLASTIN TIME (SEC)**				
FWB	15.2 (13.5–16.9)	14.2 (12.2–16.1)	14.8 (12.9–16.6)^*^, ^+^, ^#^	14.8 (13.6–16.0)^+^
CRYST	15.4 (14.1–16.6)	14.7 (12.3–17.0)	23.5 (18.2–28.8)^&^	18.8 (14.7–22.8)
HES	16.0 (14.1–17.8)	14.5 (12.2–16.8)	25.6 (21.0–30.1)^&^, ^#^	19.8 (16.7–22.9)^&^
GELO	15.2 (14.1–16.3)	14.1 (12.8–15.4)	21.0 (16.5–25.5)^&^, ^#^	18.5 (16.7–20.2)
**FIBRINOGEN CONCENTRATION (g/L**^−1^**)**				
FWB	1.33 (1.07–1.59)	0.93 (0.73–1.13)	0.99 (0.80–1.19)*, +, #	0.93 (0.74–1.12)
CRYST	1.41 (1.19–1.62)	0.93 (0.76–1.09)	0.52 (0.42–0.62)^&^	0.60 (0.46–0.74)
HES	1.42 (1.02–1.81)	0.94 (0.58–1.29)	0.51 (0.33–0.69)^&^	0.64 (0.46–0.82)
GELO	1.51 (1.16–1.85)	1.11 (0.82–1.39)	0.64 (0.42–0.87)^&^	0.67 (0.41–0.93)
**FACTOR VII ACTIVITY (%)**				
FWB	102.6 (87.9–117.4)	79.9 (66.7–93.1)	78.8 (70.7–87.0)*, +, #	72.5 (63.9–81.1)^+^, ^#^
CRYST	108.5 (87.6–129.4)	83.8 (65.1–102.5)	55.3 (42.8–67.7)^&^	62.6 (45.4–79.8)
HES	90.2 (77.1–103.3)	70.1 (52.7–87.5)	45.3 (34.6–56.1)^&^	50.7 (39.9–61.5)^&^
GELO	95.1 (83.0–107.3)	77.5 (66.3–88.7)	53.0 (45.2–60.8)^&^	52.6 (43.5–61.7)^&^
**FACTOR VIII ACTIVITY (%)**				
FWB	127.5 (86.9–168.2)	153.2 (131.8–174.6)	138.6 (110.6–166.6)*, +, #	117.4 (106.4–128.3)
CRYST	108.8 (81.8–135.9)	169.0 (121.8–216.3)	71.2 (54.9–87.4)^&^	93.6 (64.9–122.2)
HES	93.8 (77.8–109.9)	162.0 (107.5–216.5)	75.4 (61.1–89.7)^&^	83.3 (66.5–100.1)
GELO	109.1 (85.2–133.0)	137.5 (116.4–158.6)	83.3 (56.3–110.3)^&^	86.3 (66.0–106.6)
**VON WILLEBRAND FACTOR ANTIGEN (%)**				
FWB	103.5 (77.0–130.0)	71.8 (35.0–108.5)	96.7 (64.8–128.6)*, +, #	82.1 (44.3–120.0)
CRYST	102.5 (74.2–130.7)	86.7 (46.6–126.9)	42.2 (31.9–52.4)^&^	48.5 (30.2–66.8)
HES	91.5 (78.5–104.5)	61.6 (30.2–93.1)	47.1 (33.4–60.9)^&^	51.8 (34.3–69.3)
GELO	105.1 (67.5–142.7)	77.0 (41.8–112.2)	53.9 (35.8–72.0)^&^	41.8 (19.9–63.8)

*Fluid was delivered directly after shock time point*.

& Significantly different to FWB;

* Significantly different to CRYST;

+ Significantly different to HES;

# *Significantly different to GELO (Bonferroni–Holm corrected P-value < 0.05)*.

### Comparisons between baseline and shock (all dogs)

For the secondary objective, hemorrhagic shock was associated with platelet activation and several markers of hypocoagulability, compared with baseline (Table 5). There was a significantly shorter PCT, with no significant difference in platelet count. Evidence of hypocoagulability on ROTEM included significantly longer EXTEM CT, INTEM CFT, and EXTEM CFT, and significantly lower INTEM MCF, EXTEM MCF, FIBTEM MCF, and EXTEM TPI. Conversely, there was significantly shorter INTEM CT. There was also evidence of hyperfibrinolysis, with significantly lower EXTEM LI60. There was a significant difference in frequencies of hyperfibrinolysis scores between baseline and shock (*P* = 0.0145). At baseline, 23 dogs showed no hyperfibrinolysis and 1 showed irreversible hyperfibrinolysis; at shock, 15 dogs showed no hyperfibrinolysis, 4 showed reversible hyperfibrinolysis, 4 showed irreversible hyperfibrinolysis, and 1 showed paradoxical increase in hyperfibrinolysis. Evidence of hypocoagulability on plasma coagulation assays included significantly longer PT and significantly lower fibrinogen concentration, FVII and vWF. Conversely, there was significantly shorter APTT and significantly higher FVIII.

**Table 5:** Platelet closure time and count, rotational thromboelastometry, and plasma coagulation assay parameters (mean, 95% confidence interval) in dogs (*n* = 24) with hemorrhagic shock.

**Parameter**	**Baseline**	**Shock**	*P* value^*^
Platelet Closure Time (sec)	83.4 (77.6–89.1)	69.0 (65.2–72.8)	**<0.001**
Estimated Platelet Count (x10^9^ L^−1^)	134.3 (124.7–143.9)	119.4 (106.3–132.5)	0.057
INTEM CT (sec)	218.3 (180.9–255.7)	168.1 (143.2–193.0)	**<0.001**
INTEM CFT (sec)	158.5 (135.9–181.1)	234.6 (203.6–265.6)	**<0.001**
INTEM α (°)	61.0 (57.4–64.5)	58.6 (55.3–61.9)	0.067
INTEM MCF (mm)	52.4 (50.5–54.3)	47.4 (45.4–49.4)	**<0.001**
INTEM LI60 (%)	99.3 (98.9–99.8)	99.1 (98.6–99.7)	0.458
EXTEM CT (sec)	54.3 (47.3–61.2)	66.5 (60.1–73.0)	**<0.001**
EXTEM CFT (sec)	148.0 (125.3–170.7)	193.6 (170.5–216.8)	**<0.001**
EXTEM α (°)	63.4 (61.2–65.6)	62.2 (60.5–63.8)	0.117
EXTEM MCF (mm)	52.2 (50.0–54.4)	45.7 (43.0–48.4)	**<0.001**
EXTEM LI60 (%)	96.0 (91.4–100.7)	84.1 (74.9–93.3)	**0.002**
EXTEM TPI	24.1 (19.8–28.4)	15.5 (11.8–19.2)	**<0.001**
FIBTEM MCF (mm)	5.0 (4.2–5.9)	3.5 (2.6–4.4)	**<0.001**
Prothrombin Time (sec)	7.5 (7.3–7.7)	8.5 (8.2–8.8)	**<0.001**
Activated Partial Thromboplastin Time (sec)	15.4 (14.8–16.0)	14.4 (13.6–15.1)	**<0.001**
Fibrinogen Concentration (g L^−1^)	1.41 (1.29–1.54)	0.97 (0.87–1.08)	**<0.001**
Factor VII Activity (%)	99.1 (92.5–105.7)	77.8 (71.7–84.0)	**<0.001**
Factor VIII Activity (%)	109.8 (98.0–121.6)	155.4 (139.9–171.0)	**<0.001**
von Willebrand Factor Antigen (%)	100.6 (90.0–111.3)	74.3 (60.2–88.4)	**<0.001**

* *Paired t-test, bold indicates P < 0.05. α, alpha angle; CFT, clot formation time; CT, clotting time; LI60, lysis index at 60 min; MCF, maximum clot firmness; TPI, thrombodynamic potential index*.

## Discussion

The main finding of our study was evidence of enhanced hypocoagulability with administration of HES, compared with GELO, FWB, and CRYST, as indicated by multiple ROTEM parameters and plasma coagulation assays. Additionally, GELO administration significantly impaired platelet function, as measured by PCT, compared with all other fluid groups. These differences were beyond the effects of hemodilution, as indicated by no significant differences in hemoglobin concentration or platelet count between the HES, GELO, and CRYST groups. As a secondary objective, this study also provides evidence of hypocoagulability and platelet hyperreactivity in dogs with atraumatic hemorrhagic shock.

Dogs administered HES in our study showed evidence of hypocoagulability beyond hemodilution on ROTEM, most notably having a longer EXTEM CT compared with all other groups. A similar prolongation of EXTEM CT was seen in a previous *in vitro* study where canine blood was diluted with HES 130/0.4 (16). EXTEM CT is primarily affected by extrinsic and common pathway coagulation factors (31). However, no difference was detected in FVII between HES, GELO, and CRYST, and other coagulation tests involving the common pathway, such as INTEM CT, PT, and APTT, did not appear to be affected to the same extent. Therefore, the mechanism of the prolongation of EXTEM CT is unclear. The INTEM and EXTEM MCF were also lower with HES, compared with FWB, similar to when canine blood was diluted with HES 130/0.4 *in vitro* (16). INTEM and EXTEM MCF are primarily affected by platelet function, functional fibrinogen and factor XIII (31, 32). Fibrinogen concentration was significantly lower in HES, compared with FWB, which likely contributed.

The changes in EXTEM CT, INTEM MCF, and EXTEM MCF with HES at T60 were beyond the limits of our canine institutional reference intervals (EXTEM CT: 30–94 s, INTEM MCF: 42–67 mm, EXTEM MCF: 42–70 mm), however the clinical relevance of these abnormalities in dogs is unknown. Also, note that our institutional reference intervals were established with venous blood from conscious non-greyhound dogs. Prolongation of EXTEM CT is predictive of hemorrhage in humans undergoing liver transplantation (33). Reduction in MCF is predictive of blood loss and transfusion requirements in human trauma and surgical patients (34–36). In dogs, EXTEM CT was longer and EXTEM MCF was lower in cases that had hemorrhage, compared to those that did not, with *Angiostrongylus* vasorum infection (37) and leptospirosis (38). Therefore, the effect of HES on coagulation may contribute to risk of hemorrhage in the dog. Clinical studies are needed to further characterize coagulation dysfunction with HES 130/0.4 in the dog. However, it is prudent to use HES with caution in dogs at risk of significant hemorrhage, and evaluation of coagulation using viscoelastic tests should be considered in dogs showing clinical signs of hemorrhage following HES administration.

There was evidence of impaired platelet function following GELO administration in our study, as measured by a higher PCT. This has not been previously reported in dogs. In humans, GELO has been associated with prolongation of bleeding time (39) and PCT (40), decreased integrin α_IIb_β_3_ activity (40) and decreased ristocetin-stimulated platelet aggregation (39, 41). Pigs administered GELO showed decreased aggregation on collagen-stimulated impedance aggregometry (42). Binding of GELO molecules to the platelet surface, including receptors such as integrin α_IIb_β_3_, is a potential mechanism. In our study, mean PCT in the GELO group was prolonged beyond our non-greyhound canine institutional reference interval (52–101 s), as well as the published reference interval in greyhounds (43). The clinical relevance of PCT prolongation in dogs is not fully known, however there is a weak correlation between PCT and clinical bleeding in dogs with congenital von Willebrand Disease (44). Clinical studies to assess risk of hemorrhage in dogs treated with GELO are needed, and GELO should be used with caution in dogs at risk of significant hemorrhage or with other disturbances of coagulation. Conversely, HES did not show evidence of platelet dysfunction in our study. This is similar to a previous greyhound hemorrhagic shock model (10) but differs from a conscious healthy dog study that used HES 670/0.75 (18). Hydroxyethyl starch products with lower MW and degree of substitution have less effect on platelet function *in vitro* in dog blood (13) and *in vivo* in people (45). Clinical studies are needed to further evaluate platelet dysfunction with HES 130/0.4 in the dog, as this may be affected by dose, duration of therapy and comorbidities, however this study does not support clinically relevant platelet antagonism.

A secondary exploratory objective of this study was to assess coagulation before and after hemorrhagic shock in all subjects. There was evidence of platelet hyperreactivity with hemorrhagic shock in our study, with a significant decrease in PCT. A similar decrease in PCT that was not statistically significant was seen in a previous greyhound hemorrhagic shock model study (27), where the lack of significance was postulated to be a type 2 error due to the small sample size. In contrast, another canine hemorrhagic shock model study (9) in beagles showed decreased platelet function, as measured by impedance aggregometry. The different findings may reflect differences between PCT and aggregometry, differences between breeds of dog or differences in the severity and duration of shock, with more severe hypotension seen in the latter study. In one study of people with significant intraoperative hemorrhage, platelet function measured by impedance aggregometry initially decreased, then increased above baseline (46), suggesting duration of shock may affect platelet activation. Results of ROTEM and plasma coagulation assays showed evidence of decreased coagulation factor activity, delayed clot formation, impaired clot strength and increased fibrinolysis during hemorrhagic shock. There were also some markers of hypercoagulability, notably decreases in INTEM CT and APTT and an increase in FVIII, highlighting the complexity of coagulopathy in shock. These findings were more pronounced than a previous canine hemorrhagic shock model (9), which may be due to a larger sample size in the current study, differences between breeds of dog or differences in the severity and duration of shock. Our findings were similar to dogs with spontaneous hemoperitoneum (8), which were also hypocoagulable and hyperfibrinolytic. There were also similarities with some studies of dogs with traumatic hemorrhagic shock (6, 7), however it should be noted that traumatic hemorrhagic shock may differ from atraumatic hemorrhagic shock due to injury-induced endothelial activation, auto-heparinization and inflammation (47). Also, the dogs in this study were under general anesthesia and receiving continuous crystalloid infusion, which may have also altered coagulation. Larger clinical studies of coagulation impairment in dogs with naturally-occurring hemorrhagic shock are needed.

The major limitation of our study was the use of ex-racing greyhounds, which are not purpose-bred or housed in a laboratory environment. These dogs were unable to be rehomed and were donated for blood product collection for clinical use, anatomy cadaver teaching and other research. Their use in research immediately before euthanasia obviates procurement of other large animals purely for research purposes. Greyhounds are known to have differences in coagulation compared with other breeds (48), notably including a syndrome of delayed postoperative bleeding that may be due to excessive fibrinolysis (49, 50). Interestingly, in our study there was minimal evidence of hyperfibrinolysis, based on ROTEM LI60. However, there is little published data on how to interpret fibrinolysis parameters on standard viscoelastic tests in the dog (28). Previous studies using thromboelastography have also failed to show differences in fibrinolysis parameters between greyhounds and other dog breeds (48). In our study the mean FIBTEM MCF at baseline was 5.0 mm, which is below the previously published normal reference interval for dogs (16). Whilst breed differences may have impacted the absolute results of this study, our primary objective was to compare the effects of different fluids, and the relative changes are still highly relevant. Most studies of coagulation use venous blood, however arterial blood was used in this study for practical reasons. The clinical relevance of alterations in ROTEM parameters is not fully understood. Finally, our study was limited by sample size, which may have contributed to type 2 error and lack of finding further differences. Insufficient data was available for power calculation for many of the parameters tested.

## Conclusion

Our study showed that in dogs with hemorrhagic shock, volume replacement with FWB was associated with the least impairment of coagulation compared with HES, GELO, and CRYST. Both GELO and HES impaired coagulation beyond the effects of hemodilution, with GELO showing impaired platelet function and HES showing hypocoagulable ROTEM and plasma coagulation assays. Clinical trials to assess the risk of coagulation impairment and bleeding in dogs with naturally-occurring hemorrhagic shock treated with synthetic colloids are needed. Our study provides evidence that HES and GELO should be used with caution in dogs at risk of significant hemorrhage.

## Data availability statement

The raw data supporting the conclusions of this manuscript will be made available by the authors, without undue reservation, to any qualified researcher.

## Author contributions

CB contributed to design of the experiment, data collection, laboratory work, interpretation of results, and wrote the manuscript. MC contributed to design of the experiment, data collection, laboratory work, and reviewed the manuscript. AR contributed to design of the experiment, data collection, and reviewed the manuscript. GH contributed to design of the experiment, performed the statistical analysis, and reviewed the manuscript. CS contributed to laboratory work and reviewed the manuscript. LS contributed to design of the experiment, data collection, laboratory work, interpretation of results, and reviewed the manuscript. All authors read and approved the final manuscript.

### Conflict of interest statement

The authors declare that the research was conducted in the absence of any commercial or financial relationships that could be construed as a potential conflict of interest.
