# Corrigendum: Hypocoagulability and Platelet Dysfunction Are Exacerbated by Synthetic Colloids in a Canine Hemorrhagic Shock Model

**DOI:** 10.3389/fvets.2020.00641

**Published:** 2020-09-08

**Authors:** Corrin J. Boyd, Melissa A. Claus, Anthea L. Raisis, Giselle Hosgood, Claire R. Sharp, Lisa Smart

**Affiliations:** School of Veterinary and Life Sciences, College of Veterinary Medicine, Murdoch University, Perth, WA, Australia

**Keywords:** hydroxyethyl starch, succinylated gelatin, crystalloid, fresh whole blood, platelet closure time, PFA-100, rotational thromboelastometry (ROTEM), viscoelastic coagulation tests

In the original article, there was a mistake in Table 2 as published. **The EXTEM CFT baseline mean (95% confidence interval) for the HES group was slightly incorrect due to a data entry error**. The corrected Table 2 appears below.

**Table 2:** Rotational thromboelastometry parameters (mean, 95% confidence interval) in dogs (*n* = 6 per group) with hemorrhagic shock given 20 mL kg^−1^ of either fresh whole blood (FWB), hydroxyethyl starch 130/0.4 (HES), 4% succinylated gelatin (GELO), or 80 mL kg^−1^ of balanced isotonic crystalloid (CRYST).

**Parameter**	**Baseline**	**Shock**	**T60**	**T180**
**INTEM CT (sec)**				
FWB	279.3 (137.5–421.2)	204.8 (123.1–286.5)	330.5 (17.2–643.8)	269.0 (150.7–387.3)
CRYST	171.3 (149.1–193.5)	160.7 (129.2–192.1)	162.2 (134.0–190.3)	159.8 (142.8–176.9)
HES	201.2 (129.1–273.2)	145.8 (72.2–219.5)	231.8 (130.4–333.3)	199.0 (113.0–285.0)
GELO	221.5 (146.0–297.0)	161.0 (117.0–205.0)	224.3 (108.6–340.1)	193.5 (146.5–240.5)
**INTEM CFT (sec)**				
FWB	224.2 (143.1–305.2)	260.3 (180.6–340.0)	303.8 (102.0–505.7)	235.3 (178.9–291.8)
CRYST	143.5 (119.1–167.9)	227.8 (190.9–264.8)	298.8 (269.5–328.1)	234.5 (207.6–261.4)
HES	202.8 (106.9–298.8)	264.0 (168.0–360.0)	431.5 (287.3–575.7)	309.2 (221.0–397.3)
GELO	178.2 (128.4–227.9)	186.2 (114.2–258.1)	305.5 (211.1–399.9)	278.7 (178.1–379.2)
**INTEM** **α** **(°)**				
FWB	54.2 (44.9–63.4)	54.0 (46.3–61.7)	52.7 (38.5–66.8)	56.2 (49.8–62.6)
CRYST	68.0 (65.4–70.6)	62.7 (57.2–68.1)	56.8 (53.7–60.0)	60.7 (59.8–61.5)
HES	60.5 (49.4–71.6)	56.0 (46.5–65.5)	50.7 (40.2–61.1)	53.5 (46.4–60.6)
GELO	61.2 (56.5–65.9)	61.7 (53.8–69.6)	53.0 (46.7–59.3)	56.2 (49.8–62.6)
**INTEM MCF (mm)**				
FWB	51.2 (46.9–55.4)	47.7 (43.0–52.3)	45.3 (38.3–52.3)^+^	48.2 (44.5–51.8)^+^
CRYST	54.2 (50.8–57.5)	46.3 (43.0–49.7)	42.2 (39.9–44.4)	44.0 (41.4–46.6)
HES	50.3 (45.3–55.4)	44.5 (40.3–48.7)	37.0 (31.6–42.4)^&^	41.0 (36.2–45.8)^&^
GELO	54.0 (48.3–59.7)	51.2 (45.8–56.5)	41.5 (36.0–47.0)	42.8 (37.2–48.5)
**INTEM LI60 (%)**				
FWB	99.0 (97.4–100.6)	99.7 (98.8–100.5)	99.7 (98.8–100.5)	99.2 (97.9–100.4)
CRYST	99.7 (98.8–100.5)	98.7 (97.6–99.8)	96.8 (94.7–99.0)	97.2 (94.4–99.9)
HES	99.2 (97.9–100.4)	98.5 (96.4–100.6)	87.8 (67.3–108.4)	90.0 (74.1–105.9)
GELO	99.5 (98.6–100.4)	99.7 (99.1–100.2)	98.3 (94.0–102.6)	96.5 (87.5–105.5)
**EXTEM CT (sec)**				
FWB	52.5 (40.8–64.2)	75.2 (57.2–93.1)	64.7 (45.3–84.1)^+^	62.0 (53.1–70.9)
CRYST	54.8 (37.8–71.9)	66.2 (59.4–72.9)	72.8 (62.9–82.8)^+^	71.5 (58.7–84.3)
HES	61.8 (40.6–83.0)	69.0 (56.1–81.9)	119.3 (57.4–181.3)^&^^,^^*^^,^^#^	82.7 (50.6–114.8)
GELO	47.8 (28.6–67.0)	55.8 (36.1–75.6)	76.2 (27.5–124.8)^+^	75.2 (50.6–99.8)
**EXTEM CFT (sec)**				
FWB	178.2 (121.5–234.8)	217.3 (149.5–285.2)	251.0 (127.6–374.4)	202.2 (148.5–255.9)
CRYST	129.3 (115.4–143.3)	184.2 (152.6–215.7)	246.0 (198.3–293.7)	221.0 (177.7–264.3)
HES	187.2 (106.0–268.3)	208.0 (137.8–278.2)	373.4 (179.9–566.9)	437.3 (0.0–918.6)
GELO	139.3 (102.6–176.1)	165.0 (115.5–214.5)	230.4 (167.7–293.1)	263.3 (174.5–352.1)
**EXTEM** **α** **(°)**				
FWB	59.8 (53.5–66.1)	60.0 (55.9–64.1)	55.8 (48.8–62.9)	58.3 (54.4–62.3)
CRYST	66.5 (64.4–68.6)	63.3 (60.9–65.8)	59.3 (54.2–64.5)	60.8 (59.0–62.6)
HES	61.7 (55.1–68.2)	61.8 (56.4–67.2)	46.2 (28.6–63.8)	53.0 (39.8–66.2)
GELO	65.7 (62.8–68.5)	63.5 (59.9–67.1)	49.5 (32.7–66.3)	56.0 (52.5–59.5)
**EXTEM MCF (mm)**				
FWB	50.5 (46.3–54.7)	44.7 (39.6–49.7)	45.3 (38.6–52.1)^+^	47.0 (43.4–50.6)
CRYST	54.7 (52.1–57.2)	45.7 (42.1–49.3)	39.0 (33.3–44.7)	39.3 (34.7–44.0)
HES	49.3 (42.7–55.9)	42.5 (33.4–51.6)	31.2 (20.5–41.9)^&^	37.8 (28.6–47.1)
GELO	54.3 (47.8–60.8)	50.0 (43.4–56.6)ß	39.0 (26.6–51.4)	40.3 (31.9–48.8)
**EXTEM TPI**				
FWB	18.8 (12.7–25.0)	12.5 (6.7–18.3)	12.3 (5.6–19.0)	14.0 (9.6–18.4)
CRYST	28.5 (22.8–34.2)	14.3 (10.5–18.2)	8.2 (5.6–10.8)	9.3 (6.1–12.6)
HES	20.2 (7.4–32.9)	14.2 (1.1–27.3)	4.8 (2.0–7.6)	8.2 (1.4–14.9)
GELO	28.8 (15.3–42.4)	21.0 (10.3–31.7)	11.2 (5.3–17.1)	9.5 (4.2–14.8)
**EXTEM LI60 (%)**				
FWB	89.3 (66.9–111.7)	78.7 (48.1–109.3)	90.7 (70.7–110.7)	91.0 (78.2–103.8)
CRYST	99.0 (97.9–100.1)	87.7 (73.0–102.3)	84.7 (52.9–116.5)	78.8 (58.5–99.2)
HES	98.3 (95.5–101.2)	83.3 (56.1–110.6)	74.3 (36.5–112.2)	84.3 (56.2–112.5)
GELO	97.5 (93.8–101.2)	86.7 (65.3–108.1)	75.8 (49.2–102.5)	72.5 (42.5–102.5)
**FIBTEM MCF (mm)**				
FWB	4.3 (3.5–5.2)	3.5 (2.4–4.6)	3.3 (1.5–5.2)	2.8 (1.0–4.6)
CRYST	6.0 (3.1–8.9)	4.7 (1.9–7.5)	2.8 (1.0–4.6)	3.2 (1.2–5.1)
HES	5.0 (2.6–7.4)	2.0 (0.0–4.9)	0.7 (0.0–2.4)	2.2 (0.0–5.2)
GELO	4.8 (3.3–6.4)	3.8 (2.3–5.4)	2.0 (0.2–3.8)	2.7 (1.1–4.2)

*Fluid was delivered directly after Shock time point*.

& *Significantly different to FWB*;

* *Significantly different to CRYST*;

+ *Significantly different to HES*;

# *Significantly different to GELO (Bonferroni–Holm corrected P-value < 0.05). α, alpha angle; CFT, clot formation time; CT, clotting time; LI60, lysis index at 60 min; MCF, maximum clot firmness; TPI, thrombodynamic potential index*.

In the original article, there was a mistake in Table 5 as published. **The EXTEM CFT baseline mean (95% confidence interval) was slightly incorrect due to a data entry error**. The corrected Table 5 appears below.

**Table 5:** Platelet closure time and count, rotational thromboelastometry, and plasma coagulation assay parameters (mean, 95% confidence interval) in dogs (*n* = 24) with hemorrhagic shock.

**Parameter**	**Baseline**	**Shock**	*P* value^*^
Platelet Closure Time (sec)	83.4 (77.6–89.1)	69.0 (65.2–72.8)	**<0.001**
Estimated Platelet Count (x10^9^ L^−1^)	134.3 (124.7–143.9)	119.4 (106.3–132.5)	0.057
INTEM CT (sec)	218.3 (180.9–255.7)	168.1 (143.2–193.0)	**<0.001**
INTEM CFT (sec)	158.5 (135.9–181.1)	234.6 (203.6–265.6)	**<0.001**
INTEM α (°)	61.0 (57.4–64.5)	58.6 (55.3–61.9)	0.067
INTEM MCF (mm)	52.4 (50.5–54.3)	47.4 (45.4–49.4)	**<0.001**
INTEM LI60 (%)	99.3 (98.9–99.8)	99.1 (98.6–99.7)	0.458
EXTEM CT (sec)	54.3 (47.3–61.2)	66.5 (60.1–73.0)	**<0.001**
EXTEM CFT (sec)	148.0 (125.3–170.7)	193.6 (170.5–216.8)	**<0.001**
EXTEM α (°)	63.4 (61.2–65.6)	62.2 (60.5–63.8)	0.117
EXTEM MCF (mm)	52.2 (50.0–54.4)	45.7 (43.0–48.4)	**<0.001**
EXTEM LI60 (%)	96.0 (91.4–100.7)	84.1 (74.9–93.3)	**0.002**
EXTEM TPI	24.1 (19.8–28.4)	15.5 (11.8–19.2)	**<0.001**
FIBTEM MCF (mm)	5.0 (4.2–5.9)	3.5 (2.6–4.4)	**<0.001**
Prothrombin Time (sec)	7.5 (7.3–7.7)	8.5 (8.2–8.8)	**<0.001**
Activated Partial Thromboplastin Time (sec)	15.4 (14.8–16.0)	14.4 (13.6–15.1)	**<0.001**
Fibrinogen Concentration (g L^−1^)	1.41 (1.29–1.54)	0.97 (0.87–1.08)	**<0.001**
Factor VII Activity (%)	99.1 (92.5–105.7)	77.8 (71.7–84.0)	**<0.001**
Factor VIII Activity (%)	109.8 (98.0–121.6)	155.4 (139.9–171.0)	**<0.001**
von Willebrand Factor Antigen (%)	100.6 (90.0–111.3)	74.3 (60.2–88.4)	**<0.001**

* *Paired t-test, bold indicates P < 0.05. α, alpha angle; CFT, clot formation time; CT, clotting time; LI60, lysis index at 60 min; MCF, maximum clot firmness; TPI, thrombodynamic potential index*.

In the original article, there was an error. **A conversion factor was inadvertently omitted from the equation for thrombodynamic potential index (TPI)**.

A correction has been made to ***Materials and Methods***, ***Coagulation Parameters***, ***Paragraph 3***:

Rotational thromboelastometry (ROTEM^®^ *delta*, Tem International GmbH) was performed according to the manufacturer's instructions and PROVETS guidelines (28) using the INTEM (star-TEM and in-TEM reagents), EXTEM (star-TEM and r ex-TEM reagents), FIBTEM (r ex-TEM and fib-TEM reagents), and APTEM (r ex-TEM and ap-TEM reagents) profiles. Measurement was started 30 min after sample collection. Each profile was run for at least 1 h following initiation. Data recorded for the INTEM and EXTEM profiles included clotting time (CT), clot formation time (CFT), alpha angle (α), maximum clot firmness (MCF), and lysis index at 60 min (LI60). Thrombodynamic potential index (TPI) was recorded for the EXTEM profile as a measure of global coagulation (29), calculated using the equations:

$$\begin{array}{l} \text{ TPI}=\text{EMX}*30/\text{CFT},\\ \text{EMX}=\left(100*\text{MCF}\right)/\left(100-\text{MCF}\right). \end{array}$$

The authors apologize for this error and state that this does not change the scientific conclusions of the article in any way. The original article has been updated.

